# (3R*,4R*)-1-(4-Chloro­phen­yl)-4-[2-hy­droxy-3-(morpholinomethyl)­phen­yl]-3-phen­oxy­azetidin-2-one

**DOI:** 10.1107/S1600536811000675

**Published:** 2011-01-12

**Authors:** Mehmet Akkurt, Aliasghar Jarrahpour, Hashem Sharghi, Seid Ali Torabi Badrabady, Orhan Büyükgüngör

**Affiliations:** a Department of Physics, Faculty of Sciences, Erciyes University, 38039 Kayseri, Turkey; b Department of Chemistry, College of Sciences, Shiraz University, 71454 Shiraz, Iran; c Department of Physics, Faculty of Arts and Sciences, Ondokuz Mayıs University, 55139 Samsun, Turkey

## Abstract

The β-lactam ring of the title compound, C_26_H_25_ClN_2_O_4_, is nearly planar (r.m.s. deviation = 0.025 Å) and the morpholine ring adopts a chair conformation. The mean plane of the β-lactam ring makes dihedral angles of 21.6 (4), 84.4 (4) and 33.7 (4)° with the two benzene rings and the phenyl ring, respectively. The conformation of the title compound is stabilized by intra­molecular C—H⋯O and O—H⋯N inter­actions. The crystal structure features C—H⋯π and aromatic π–π stacking inter­actions [centroid–centroid distances = 3.684 (4) and 3.883 (4) Å].

## Related literature

For a related structure, see: Akkurt *et al.* (2011 ▶). For puckering parameters, see: Cremer & Pople (1975 ▶).
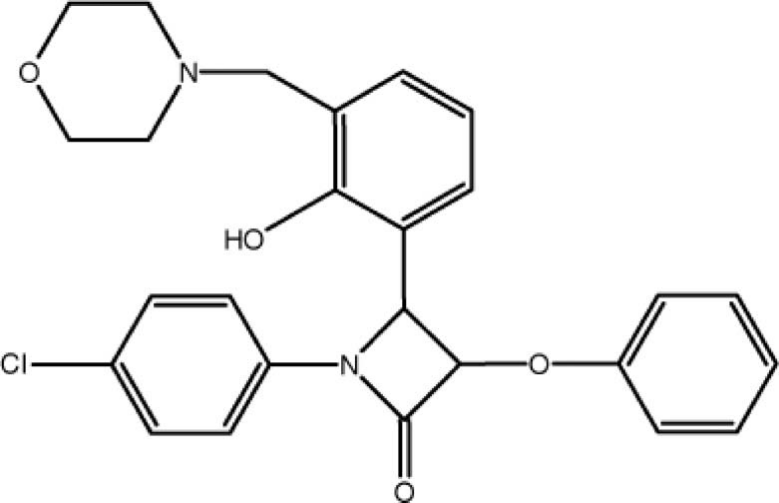


## Experimental

### 

#### Crystal data

C_26_H_25_ClN_2_O_4_
*M* *_r_* = 464.93
Monoclinic, 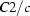

*a* = 29.6418 (18) Å
*b* = 6.7166 (3) Å
*c* = 28.5708 (15) Å
β = 123.043 (4)°
*V* = 4768.2 (5) Å^3^
*Z* = 8
Mo *K*α radiation
μ = 0.20 mm^−1^
*T* = 296 K
0.53 × 0.23 × 0.04 mm

#### Data collection

Stoe IPDS 2 diffractometer
Absorption correction: integration (*X-RED32*; Stoe & Cie, 2002 ▶) *T*_min_ = 0.904, *T*_max_ = 0.992
16824 measured reflections
4707 independent reflections
1712 reflections with *I* > 2σ(*I*)
*R*_int_ = 0.159

#### Refinement

*R*[*F* ^2^ > 2σ(*F* ^2^)] = 0.087
*wR*(*F* ^2^) = 0.130
*S* = 1.00
4707 reflections
302 parameters
1 restraint
H atoms treated by a mixture of independent and constrained refinement
Δρ_max_ = 0.15 e Å^−3^
Δρ_min_ = −0.15 e Å^−3^

### 

Data collection: *X-AREA* (Stoe & Cie, 2002 ▶); cell refinement: *X-AREA*; data reduction: *X-RED32* (Stoe & Cie, 2002 ▶); program(s) used to solve structure: *SIR97* (Altomare *et al.*, 1999 ▶); program(s) used to refine structure: *SHELXL97* (Sheldrick, 2008 ▶); molecular graphics: *ORTEP-3* (Farrugia, 1997 ▶); software used to prepare material for publication: *WinGX* (Farrugia, 1999 ▶).

## Supplementary Material

Crystal structure: contains datablocks global, I. DOI: 10.1107/S1600536811000675/hb5782sup1.cif

Structure factors: contains datablocks I. DOI: 10.1107/S1600536811000675/hb5782Isup2.hkl

Additional supplementary materials:  crystallographic information; 3D view; checkCIF report

## Figures and Tables

**Table 1:** Hydrogen-bond geometry (Å, °) *Cg*5 is a centroid of the C16–C21 benzene ring.

*D*—H⋯*A*	*D*—H	H⋯*A*	*D*⋯*A*	*D*—H⋯*A*
O3—H3*A*⋯N2	0.85 (6)	1.87 (6)	2.642 (8)	150 (5)
C2—H2⋯O2	0.93	2.50	3.304 (6)	144
C15—H15⋯O2	0.93	2.54	3.148 (7)	123
C20—H20⋯*Cg*5^i^	0.93	2.88	3.596 (5)	134

Symmetry code: (i) 
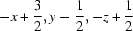
.

**Fig. 1.**
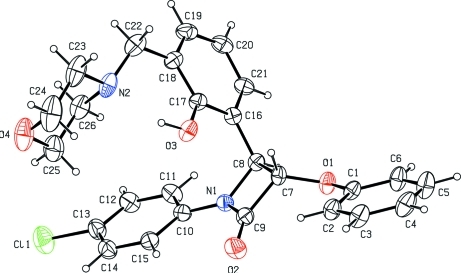
The molecular structure of (I) with displacement ellipsoids for non-H atoms drawn at the 20% probability level.

**Fig. 2.**
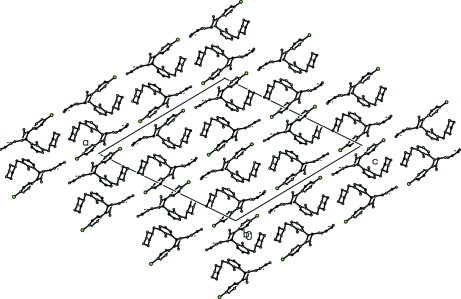
The packing of (I), viewing down the b axis. All H atoms are omitted for clarity.

## supplementary crystallographic information

### Comment

As part of our ongoing structural studies of β-lactams (Akkurt *et al.*, 2011), we now report the structure of the title compound, (I).

In the title compound, (I), (Fig. 1), the β-lactam ring (N1/C7–C9) is almost planar, with long C—C distances [C7–C9 = 1.518 (7) Å and C7–C8 = 1.553 (6) Å]. The morpholine ring (N2/O4/C23—C26) adopts a chair conformation, with the puckering parameters of Q_T_ = 0.594 (8) Å, θ = 177.1 (8)° and φ = 305 (17)° (Cremer & Pople, 1975). The dihedral angles between the mean planes of the rings in (I) are given in Table 2.

The molecular conformation of (I) is stabilized by intra-molecular C— H···O and O— H···N interactions (Table 1). The crystal structure is stabilized by C—H&ctdot;π interactions (Table 1) and two π-π stacking interactions [*Cg*1···*Cg*5(*x*, *y*, *z*) = 3.684 (4) Å and *Cg*4···*Cg*4(1 - *x*, -*y*, -*z*) = 3.883 (4) Å, where *Cg*1, *Cg*4 and *Cg*5 are the centroids of the N1/C7–C9 β-lactam, the C10–C15 and C16–C21 benzene rings, respectively]. Fig. 2 shows the packing of (I), down the *b* axis.

### Experimental

To a solution of (*E*)-2-((4-chlorophenylimino)methyl)-6-(morpholinomethyl)phenol (1.0 mmol) and triethylamine (2.6 mmol) in dry CH_2_Cl_2_ was slowly added phenoxyacetyl chloride (1.3 mmol) in dry CH_2_Cl_2_ (10 ml) at 195 K. The reaction mixture was then allowed to warm to room temperature, stirred over night and then it was washed with saturated sodium bicarbonate solution (20 ml), brine (20 ml), dried (Na_2_SO_4_). The solvent was evaporated under reduced pressure to give the crude product which was then purified by column chromatography over silica gel.  Colourless needles were recrystallised from ethyl acetate (yield 65%). [mp: 463–465 K]. IR (KBr, cm^-1^): 1758.5 (CO β-Lactam), 3311–3497 (OH). ^1^H-NMR (250 MHz, CDCl_3_) δ (p.p.m): 3.43 (CH_2_—N, t, 4H, *J* = 13.5 Hz), 3.70 (CH_2_, s, 2H), 3.88 (CH_2_—O, t, 4H, *J* = 13.9 Hz), 5.59 (H-8, d, 1H, *J* = 1.6 Hz), 5.91 (H-7, d, 1H, *J* = 1.6 Hz), 6.67–7.35 (ArH, m, 12H); ^13^C-NMR (62.9 MHz, CDCl_3_) δ (p.p.m): 52.6 (CH_2_—N), 55.9 (C-22), 50.2 (O—CH_2_), 66.5 (C-8), 80.9 (C-7), 115.2–157.0 (aromatic carbons), 163.1 (CO β-Lactam). Analysis calculated for C_26_H_25_ClN_2_O_4_: C 67.17, H 5.42, N 6.03%. found: C 67.05, H 5.47, N 6.08%.

### Refinement

The position of the bridging hydroxyl hydrogen atom was found in a difference Fourier map, and refined freely by the soft- constraint method of the hydrogen-position of 0.83 (2) Å. The other H atoms were placed at calculated positions and were treated as riding on their parent atoms with O—H = 0.82 Å, C—H = 0.93 (aromatic), 0.96(methyl), 0.97 Å (methyline) and 0.98 Å (methine), and with *U*_iso_(H) = 1.5*U*_eq_(C) for methyl and *U*_iso_(H) = 1.2*U*_eq_(C) for aromatic, methine and methyline.

### Crystal data

**Table:**

C_26_H_25_ClN_2_O_4_	*F*(000) = 1952
*M**_r_* = 464.93	*D*_x_ = 1.295 Mg m^−^^3^
Monoclinic, *C*2/*c*	Mo *K*α radiation, λ = 0.71073 Å
Hall symbol: -C 2yc	Cell parameters from 9239 reflections
*a* = 29.6418 (18) Å	θ = 1.4–26.6°
*b* = 6.7166 (3) Å	µ = 0.20 mm^−^^1^
*c* = 28.5708 (15) Å	*T* = 296 K
β = 123.043 (4)°	Needle, colourless
*V* = 4768.2 (5) Å^3^	0.53 × 0.23 × 0.04 mm
*Z* = 8	

### Data collection

**Table:**

Stoe IPDS 2 diffractometer	4707 independent reflections
Radiation source: sealed X-ray tube, 12 x 0.4 mm long-fine focus	1712 reflections with *I* > 2σ(*I*)
plane graphite	*R*_int_ = 0.159
Detector resolution: 6.67 pixels mm^-1^	θ_max_ = 26.2°, θ_min_ = 1.6°
ω scans	*h* = −36→36
Absorption correction: integration (*X-RED32*; Stoe & Cie, 2002)	*k* = −8→7
*T*_min_ = 0.904, *T*_max_ = 0.992	*l* = −34→32
16824 measured reflections	

### Refinement

**Table:**

Refinement on *F*^2^	Primary atom site location: structure-invariant direct methods
Least-squares matrix: full	Secondary atom site location: difference Fourier map
*R*[*F*^2^ > 2σ(*F*^2^)] = 0.087	Hydrogen site location: inferred from neighbouring sites
*wR*(*F*^2^) = 0.130	H atoms treated by a mixture of independent and constrained refinement
*S* = 1.00	*w* = 1/[σ^2^(*F*_o_^2^) + (0.0198*P*)^2^] where *P* = (*F*_o_^2^ + 2*F*_c_^2^)/3
4707 reflections	(Δ/σ)_max_ < 0.001
302 parameters	Δρ_max_ = 0.15 e Å^−^^3^
1 restraint	Δρ_min_ = −0.15 e Å^−^^3^

### Special details

**Table:**

Geometry. Bond distances, angles *etc*. have been calculated using the rounded fractional coordinates. All su's are estimated from the variances of the (full) variance-covariance matrix. The cell e.s.d.'s are taken into account in the estimation of distances, angles and torsion angles
Refinement. Refinement on *F*^2^ for ALL reflections except those flagged by the user for potential systematic errors. Weighted *R*-factors *wR* and all goodnesses of fit *S* are based on *F*^2^, conventional *R*-factors *R* are based on *F*, with *F* set to zero for negative *F*^2^. The observed criterion of *F*^2^ > σ(*F*^2^) is used only for calculating -*R*-factor-obs *etc*. and is not relevant to the choice of reflections for refinement. *R*-factors based on *F*^2^ are statistically about twice as large as those based on *F*, and *R*-factors based on ALL data will be even larger.

### Fractional atomic coordinates and isotropic or equivalent isotropic displacement parameters (Å^2^)

**Table:**

	*x*	*y*	*z*	*U*_iso_*/*U*_eq_	
Cl1	0.55205 (8)	−0.0943 (3)	−0.11446 (7)	0.1225 (9)	
O1	0.58355 (13)	0.2225 (4)	0.21000 (14)	0.0705 (15)	
O2	0.53938 (15)	0.4640 (5)	0.09015 (15)	0.0921 (16)	
O3	0.67681 (14)	0.3208 (4)	0.13843 (16)	0.0711 (15)	
O4	0.7152 (2)	0.5197 (7)	0.0038 (2)	0.133 (3)	
N1	0.57506 (14)	0.1426 (5)	0.09621 (17)	0.0565 (16)	
N2	0.74627 (19)	0.3438 (6)	0.1079 (2)	0.080 (2)	
C1	0.58006 (18)	0.3860 (7)	0.2379 (2)	0.064 (2)	
C2	0.57298 (19)	0.5775 (7)	0.2178 (2)	0.073 (2)	
C3	0.5692 (2)	0.7320 (7)	0.2476 (3)	0.085 (3)	
C4	0.5719 (2)	0.6941 (8)	0.2963 (3)	0.097 (3)	
C5	0.5791 (2)	0.5024 (9)	0.3163 (3)	0.103 (3)	
C6	0.5825 (2)	0.3474 (7)	0.2858 (2)	0.082 (3)	
C7	0.60357 (19)	0.2554 (6)	0.1756 (2)	0.059 (2)	
C8	0.61155 (18)	0.0579 (6)	0.15272 (19)	0.0549 (17)	
C9	0.5657 (2)	0.3163 (7)	0.1150 (2)	0.064 (2)	
C10	0.56823 (18)	0.0872 (7)	0.0456 (2)	0.0600 (17)	
C11	0.58087 (19)	−0.1071 (6)	0.0384 (2)	0.071 (2)	
C12	0.5758 (2)	−0.1594 (7)	−0.0105 (2)	0.077 (2)	
C13	0.5587 (2)	−0.0228 (9)	−0.0526 (2)	0.080 (2)	
C14	0.5446 (2)	0.1665 (8)	−0.0465 (2)	0.077 (2)	
C15	0.54974 (18)	0.2211 (7)	0.0022 (2)	0.067 (2)	
C16	0.66644 (18)	0.0030 (6)	0.16733 (18)	0.0544 (16)	
C17	0.69762 (19)	0.1335 (6)	0.15820 (19)	0.0580 (17)	
C18	0.7471 (2)	0.0758 (8)	0.1678 (2)	0.067 (2)	
C19	0.7664 (2)	−0.1138 (8)	0.1899 (2)	0.078 (2)	
C20	0.7373 (2)	−0.2423 (8)	0.2011 (2)	0.077 (2)	
C21	0.6879 (2)	−0.1842 (6)	0.18915 (19)	0.0653 (19)	
C22	0.7803 (2)	0.2180 (8)	0.1572 (3)	0.093 (3)	
C23	0.7780 (3)	0.5021 (9)	0.1032 (3)	0.117 (4)	
C24	0.7411 (4)	0.6337 (10)	0.0543 (4)	0.145 (5)	
C25	0.6841 (2)	0.3629 (9)	0.0067 (3)	0.111 (3)	
C26	0.7192 (2)	0.2287 (7)	0.0555 (2)	0.088 (3)	
H2	0.57070	0.60280	0.18450	0.0890*	
H3	0.56490	0.86200	0.23460	0.1020*	
H3A	0.6940 (18)	0.370 (7)	0.125 (2)	0.093 (19)*	
H4	0.56880	0.79830	0.31580	0.1160*	
H5	0.58170	0.47660	0.34970	0.1240*	
H6	0.58640	0.21700	0.29830	0.0990*	
H7	0.63640	0.33630	0.19450	0.0700*	
H8	0.59450	−0.05380	0.15940	0.0650*	
H11	0.59270	−0.20010	0.06690	0.0840*	
H12	0.58390	−0.28860	−0.01530	0.0920*	
H14	0.53160	0.25730	−0.07570	0.0920*	
H15	0.54070	0.34970	0.00620	0.0810*	
H19	0.79970	−0.15400	0.19720	0.0940*	
H20	0.75100	−0.36680	0.21670	0.0920*	
H21	0.66790	−0.27270	0.19580	0.0780*	
H22A	0.80150	0.30230	0.18970	0.1110*	
H22B	0.80490	0.14230	0.15170	0.1110*	
H23A	0.80490	0.44350	0.09800	0.1410*	
H23B	0.79640	0.58040	0.13720	0.1410*	
H24A	0.71410	0.69200	0.05950	0.1730*	
H24B	0.76160	0.74110	0.05180	0.1730*	
H25A	0.66690	0.28600	−0.02760	0.1340*	
H25B	0.65610	0.41960	0.01050	0.1340*	
H26A	0.69750	0.12460	0.05720	0.1060*	
H26B	0.74600	0.16630	0.05070	0.1060*	

### Atomic displacement parameters (Å^2^)

**Table:**

	*U*^11^	*U*^22^	*U*^33^	*U*^12^	*U*^13^	*U*^23^
Cl1	0.1695 (18)	0.1205 (13)	0.0933 (13)	0.0114 (11)	0.0819 (13)	−0.0060 (10)
O1	0.100 (3)	0.0508 (19)	0.086 (3)	−0.0019 (17)	0.067 (2)	0.0026 (17)
O2	0.115 (3)	0.070 (2)	0.091 (3)	0.038 (2)	0.056 (2)	0.023 (2)
O3	0.089 (3)	0.0445 (18)	0.106 (3)	−0.0007 (17)	0.070 (2)	0.0045 (17)
O4	0.208 (5)	0.098 (3)	0.168 (5)	−0.024 (3)	0.150 (5)	0.006 (3)
N1	0.061 (3)	0.050 (2)	0.059 (3)	0.0081 (19)	0.033 (2)	0.006 (2)
N2	0.103 (4)	0.060 (3)	0.113 (4)	−0.023 (3)	0.083 (3)	−0.021 (3)
C1	0.067 (4)	0.059 (3)	0.084 (4)	−0.003 (2)	0.052 (3)	0.001 (3)
C2	0.088 (4)	0.060 (3)	0.092 (4)	0.001 (3)	0.062 (4)	0.007 (3)
C3	0.105 (5)	0.058 (3)	0.124 (5)	0.003 (3)	0.083 (4)	0.003 (3)
C4	0.118 (5)	0.076 (4)	0.140 (6)	−0.001 (4)	0.098 (5)	−0.017 (4)
C5	0.145 (6)	0.089 (4)	0.126 (6)	−0.001 (4)	0.107 (5)	−0.003 (4)
C6	0.117 (5)	0.062 (3)	0.107 (5)	−0.003 (3)	0.086 (4)	0.003 (3)
C7	0.074 (4)	0.051 (3)	0.067 (4)	−0.001 (2)	0.049 (3)	0.003 (2)
C8	0.060 (3)	0.044 (3)	0.064 (3)	−0.005 (2)	0.036 (3)	0.002 (2)
C9	0.066 (4)	0.058 (3)	0.072 (4)	0.004 (3)	0.041 (3)	0.010 (3)
C10	0.056 (3)	0.057 (3)	0.062 (3)	−0.002 (2)	0.029 (3)	0.005 (3)
C11	0.083 (4)	0.048 (3)	0.067 (4)	−0.001 (3)	0.032 (3)	0.000 (3)
C12	0.090 (4)	0.063 (3)	0.072 (4)	0.008 (3)	0.040 (4)	−0.009 (3)
C13	0.091 (4)	0.089 (4)	0.060 (4)	0.002 (3)	0.042 (4)	−0.004 (3)
C14	0.084 (4)	0.077 (4)	0.069 (4)	0.011 (3)	0.041 (3)	0.015 (3)
C15	0.073 (4)	0.064 (3)	0.070 (4)	0.014 (3)	0.042 (3)	0.005 (3)
C16	0.063 (3)	0.041 (2)	0.060 (3)	0.001 (2)	0.034 (3)	−0.001 (2)
C17	0.063 (3)	0.046 (3)	0.065 (3)	0.007 (2)	0.035 (3)	−0.002 (2)
C18	0.057 (4)	0.073 (3)	0.074 (4)	−0.001 (3)	0.037 (3)	−0.009 (3)
C19	0.067 (4)	0.081 (4)	0.069 (4)	0.019 (3)	0.026 (3)	−0.009 (3)
C20	0.079 (4)	0.063 (3)	0.069 (4)	0.017 (3)	0.027 (3)	0.011 (3)
C21	0.066 (4)	0.053 (3)	0.065 (3)	0.007 (3)	0.028 (3)	0.008 (2)
C22	0.084 (5)	0.093 (4)	0.118 (5)	−0.009 (4)	0.066 (4)	−0.010 (4)
C23	0.165 (7)	0.083 (4)	0.173 (7)	−0.048 (5)	0.137 (6)	−0.041 (5)
C24	0.250 (10)	0.069 (4)	0.208 (10)	−0.038 (5)	0.185 (9)	−0.017 (5)
C25	0.139 (6)	0.100 (5)	0.124 (6)	−0.009 (4)	0.091 (5)	0.015 (4)
C26	0.108 (5)	0.068 (3)	0.115 (5)	−0.014 (3)	0.078 (4)	−0.012 (4)

### Geometric parameters (Å, °)

**Table:**

Cl1—C13	1.736 (6)	C17—C18	1.392 (9)
O1—C1	1.394 (6)	C18—C22	1.516 (9)
O1—C7	1.415 (7)	C18—C19	1.398 (8)
O2—C9	1.222 (6)	C19—C20	1.376 (9)
O3—C17	1.379 (5)	C20—C21	1.367 (9)
O4—C24	1.432 (10)	C23—C24	1.504 (11)
O4—C25	1.432 (9)	C25—C26	1.502 (8)
O3—H3A	0.85 (6)	C2—H2	0.9300
N1—C9	1.374 (6)	C3—H3	0.9300
N1—C10	1.397 (7)	C4—H4	0.9300
N1—C8	1.482 (6)	C5—H5	0.9300
N2—C22	1.472 (8)	C6—H6	0.9300
N2—C23	1.473 (10)	C7—H7	0.9800
N2—C26	1.474 (7)	C8—H8	0.9800
C1—C2	1.377 (7)	C11—H11	0.9300
C1—C6	1.356 (7)	C12—H12	0.9300
C2—C3	1.385 (8)	C14—H14	0.9300
C3—C4	1.373 (10)	C15—H15	0.9300
C4—C5	1.377 (8)	C19—H19	0.9300
C5—C6	1.396 (8)	C20—H20	0.9300
C7—C9	1.518 (7)	C21—H21	0.9300
C7—C8	1.553 (6)	C22—H22A	0.9700
C8—C16	1.489 (8)	C22—H22B	0.9700
C10—C15	1.380 (7)	C23—H23A	0.9700
C10—C11	1.404 (7)	C23—H23B	0.9700
C11—C12	1.367 (7)	C24—H24A	0.9700
C12—C13	1.371 (7)	C24—H24B	0.9700
C13—C14	1.379 (8)	C25—H25A	0.9700
C14—C15	1.365 (7)	C25—H25B	0.9700
C16—C17	1.398 (8)	C26—H26A	0.9700
C16—C21	1.394 (6)	C26—H26B	0.9700
			
C1—O1—C7	117.7 (4)	C3—C2—H2	120.00
C24—O4—C25	110.1 (6)	C2—C3—H3	120.00
C17—O3—H3A	107 (3)	C4—C3—H3	120.00
C8—N1—C9	94.8 (4)	C3—C4—H4	120.00
C9—N1—C10	133.2 (4)	C5—C4—H4	120.00
C8—N1—C10	129.3 (4)	C4—C5—H5	120.00
C22—N2—C23	111.3 (5)	C6—C5—H5	120.00
C23—N2—C26	107.9 (5)	C1—C6—H6	120.00
C22—N2—C26	112.4 (4)	C5—C6—H6	120.00
O1—C1—C6	116.6 (4)	O1—C7—H7	112.00
C2—C1—C6	120.8 (5)	C8—C7—H7	112.00
O1—C1—C2	122.6 (5)	C9—C7—H7	112.00
C1—C2—C3	119.2 (5)	N1—C8—H8	111.00
C2—C3—C4	120.3 (5)	C7—C8—H8	111.00
C3—C4—C5	120.3 (6)	C16—C8—H8	111.00
C4—C5—C6	119.1 (6)	C10—C11—H11	120.00
C1—C6—C5	120.4 (5)	C12—C11—H11	120.00
O1—C7—C8	112.1 (4)	C11—C12—H12	120.00
O1—C7—C9	120.3 (5)	C13—C12—H12	120.00
C8—C7—C9	86.4 (4)	C13—C14—H14	120.00
N1—C8—C7	86.7 (3)	C15—C14—H14	120.00
N1—C8—C16	115.9 (4)	C10—C15—H15	120.00
C7—C8—C16	119.2 (4)	C14—C15—H15	120.00
O2—C9—C7	136.2 (5)	C18—C19—H19	119.00
N1—C9—C7	92.0 (4)	C20—C19—H19	119.00
O2—C9—N1	131.6 (5)	C19—C20—H20	121.00
N1—C10—C11	120.0 (4)	C21—C20—H20	120.00
N1—C10—C15	121.3 (4)	C16—C21—H21	119.00
C11—C10—C15	118.8 (5)	C20—C21—H21	119.00
C10—C11—C12	120.0 (4)	N2—C22—H22A	109.00
C11—C12—C13	120.4 (5)	N2—C22—H22B	109.00
Cl1—C13—C14	120.7 (4)	C18—C22—H22A	109.00
C12—C13—C14	119.9 (5)	C18—C22—H22B	109.00
Cl1—C13—C12	119.4 (5)	H22A—C22—H22B	108.00
C13—C14—C15	120.2 (5)	N2—C23—H23A	110.00
C10—C15—C14	120.6 (5)	N2—C23—H23B	110.00
C8—C16—C17	121.7 (4)	C24—C23—H23A	110.00
C8—C16—C21	120.8 (5)	C24—C23—H23B	110.00
C17—C16—C21	117.5 (5)	H23A—C23—H23B	108.00
O3—C17—C18	121.5 (5)	O4—C24—H24A	110.00
C16—C17—C18	121.4 (4)	O4—C24—H24B	110.00
O3—C17—C16	117.1 (5)	C23—C24—H24A	110.00
C17—C18—C19	118.1 (5)	C23—C24—H24B	110.00
C19—C18—C22	120.9 (6)	H24A—C24—H24B	108.00
C17—C18—C22	121.0 (5)	O4—C25—H25A	109.00
C18—C19—C20	121.5 (6)	O4—C25—H25B	110.00
C19—C20—C21	119.0 (5)	C26—C25—H25A	110.00
C16—C21—C20	122.3 (5)	C26—C25—H25B	109.00
N2—C22—C18	111.9 (6)	H25A—C25—H25B	108.00
N2—C23—C24	109.4 (7)	N2—C26—H26A	110.00
O4—C24—C23	109.9 (6)	N2—C26—H26B	110.00
O4—C25—C26	110.7 (6)	C25—C26—H26A	110.00
N2—C26—C25	110.2 (4)	C25—C26—H26B	109.00
C1—C2—H2	120.00	H26A—C26—H26B	108.00
			
C7—O1—C1—C2	27.8 (8)	O1—C7—C9—N1	−115.5 (4)
C7—O1—C1—C6	−153.9 (5)	C9—C7—C8—C16	119.7 (5)
C1—O1—C7—C8	173.3 (4)	C8—C7—C9—N1	−1.9 (4)
C1—O1—C7—C9	−87.5 (5)	C9—C7—C8—N1	1.7 (4)
C24—O4—C25—C26	−58.8 (8)	C7—C8—C16—C21	128.8 (5)
C25—O4—C24—C23	60.3 (11)	N1—C8—C16—C21	−129.8 (4)
C10—N1—C8—C7	161.0 (5)	N1—C8—C16—C17	48.2 (6)
C9—N1—C8—C16	−122.9 (4)	C7—C8—C16—C17	−53.2 (6)
C9—N1—C10—C11	−178.9 (6)	N1—C10—C15—C14	178.3 (6)
C10—N1—C9—C7	−159.8 (6)	N1—C10—C11—C12	−177.9 (6)
C8—N1—C10—C15	−154.4 (5)	C15—C10—C11—C12	1.3 (9)
C10—N1—C8—C16	40.0 (7)	C11—C10—C15—C14	−1.0 (9)
C8—N1—C9—C7	2.0 (5)	C10—C11—C12—C13	0.4 (9)
C8—N1—C9—O2	177.5 (7)	C11—C12—C13—Cl1	−179.4 (5)
C10—N1—C9—O2	15.7 (12)	C11—C12—C13—C14	−2.4 (10)
C9—N1—C10—C15	1.9 (10)	C12—C13—C14—C15	2.8 (10)
C8—N1—C10—C11	24.8 (9)	Cl1—C13—C14—C15	179.7 (5)
C9—N1—C8—C7	−1.9 (5)	C13—C14—C15—C10	−1.1 (9)
C23—N2—C26—C25	−58.2 (7)	C17—C16—C21—C20	−0.5 (7)
C22—N2—C23—C24	−176.7 (6)	C21—C16—C17—C18	3.2 (7)
C26—N2—C22—C18	−68.0 (7)	C8—C16—C17—O3	4.2 (6)
C26—N2—C23—C24	59.6 (8)	C21—C16—C17—O3	−177.8 (4)
C22—N2—C26—C25	178.7 (6)	C8—C16—C21—C20	177.5 (4)
C23—N2—C22—C18	171.0 (5)	C8—C16—C17—C18	−174.9 (4)
O1—C1—C6—C5	−179.8 (6)	C16—C17—C18—C19	−3.5 (7)
C6—C1—C2—C3	1.0 (9)	O3—C17—C18—C22	0.4 (7)
O1—C1—C2—C3	179.2 (6)	O3—C17—C18—C19	177.6 (4)
C2—C1—C6—C5	−1.5 (9)	C16—C17—C18—C22	179.4 (5)
C1—C2—C3—C4	−0.7 (10)	C19—C18—C22—N2	146.3 (5)
C2—C3—C4—C5	0.9 (11)	C17—C18—C22—N2	−36.7 (7)
C3—C4—C5—C6	−1.3 (11)	C22—C18—C19—C20	178.3 (5)
C4—C5—C6—C1	1.6 (10)	C17—C18—C19—C20	1.1 (7)
O1—C7—C9—O2	69.3 (9)	C18—C19—C20—C21	1.4 (7)
O1—C7—C8—N1	123.1 (4)	C19—C20—C21—C16	−1.7 (7)
C8—C7—C9—O2	−177.1 (8)	N2—C23—C24—O4	−61.4 (11)
O1—C7—C8—C16	−118.9 (5)	O4—C25—C26—N2	58.4 (7)

### Hydrogen-bond geometry (Å, °)

**Table:**

Cg5 is a centroid of the C16–C21 benzene ring.

**Table:**

*D*—H···*A*	*D*—H	H···*A*	*D*···*A*	*D*—H···*A*
O3—H3A···N2	0.85 (6)	1.87 (6)	2.642 (8)	150 (5)
C2—H2···O2	0.93	2.50	3.304 (6)	144
C15—H15···O2	0.93	2.54	3.148 (7)	123
C20—H20···Cg5^i^	0.93	2.88	3.596 (5)	134

Symmetry codes: (i) −*x*+3/2, *y*−1/2, −*z*+1/2.

### Table 2 The dihedral angles between the mean planes of the rings in (I) (°)

**Table:**

	Ring-2	Ring-3	Ring-4
Ring-1	33.7 (4)	21.6 (4)	84.4 (4)
Ring-2		13.0 (3)	63.0 (3)
Ring-3			67.6 (3)

Ring-1 : N1/C7–C9 β-lactam ring, Ring-2 : C1–C6 phenyl ring, Ring-3 : C10–C15 benzene ring, Ring-4 : C16–C21 benzene ring.
